# The role of dopamine in foraging decisions in social insects

**DOI:** 10.3389/finsc.2025.1581307

**Published:** 2025-04-17

**Authors:** Dajia Ye, J. Frances Kamhi, Deborah M. Gordon

**Affiliations:** ^1^ Department of Biology, Stanford University, Stanford, CA, United States; ^2^ Department of Psychology, Neuroscience Program, Denison University, Granville, OH, United States

**Keywords:** decision-making, foraging, insect, dopamine, ant

## Abstract

Animals often need to make decisions about whether to confront risks, and climate change is making these decisions even more critical by increasing environmental stress. Biogenic amines are crucial for modulating behavior in all animals and may contribute to behavioral adaptations to changing environments through supporting decision-making involving risk. Our review focuses on the neuromodulator dopamine in insects because of its role in risk-related behavioral choices, particularly in the context of ant foraging activity. In ants, individual decisions contribute to the collective regulation of foraging activity. We consider the role of dopamine in the regulation of collective foraging activity to manage water loss in the desert red harvester ant, *Pogonomyrmex barbatus*, in the southwest US that is undergoing severe drought. We discuss dopaminergic circuitry and its involvement in decisions about foraging risk, drawing from both the vertebrate and invertebrate literature, to outline areas of future research in the role of dopamine in collective decision-making in response to changing environmental conditions.

## 1 Introduction

Animals must make decisions about whether to confront risk in many situations (1) including predation (2–4) and longer-term exposure to environmental stress such as high temperatures (5, 6). Global climate change is altering environmental conditions for many animals, leading them to shift where they may encounter new risks. It is also driving shifts in behavioral strategies (7, 8) that alter species interactions and the ability to obtain resources (9–11). Adaptation to these challenges depends on the neural mechanisms that govern risk-related decision-making. While many neurotransmitters are likely involved in these behavioral adaptations (12, 13), here we will focus on the biogenic amine dopamine (DA), which plays a central role in regulating decision-making under risk.

DA levels in animal brains can change quickly, allowing rapid behavioral adjustment to current conditions (14). DA is synthesized and stored in dopaminergic neurons (DANs) until it is released via Ca^2+^ induced exocytosis (15), typically within milliseconds of an action potential (16, 17). It acts on DA receptors located on target cells, including downstream neurons associated with behavior (18).

DA plays a key role in evaluating potential reward and punishment, particularly in risky situations. It influences the assessment of discrepancies between expected and actual outcomes in mammals (19–21) and insects (22, 23). When an outcome is better than expected, DA levels increase, reinforcing rewarding actions (24). Conversely, when an outcome is worse than expected, DA levels decrease, discouraging the behavior (24). DA shapes decision-making and motivation by modulating how attractive or aversive different options appear (25): higher DA levels are generally associated with increased motivation to pursue rewards, whereas lower levels reduce the perceived value of potential rewards. Elevated DA levels also promote risk-seeking, while reduced DA activity encourages more cautious decision-making (26–31).

This review explores how DA influences risk-related behavioral choices, with a focus on its role in foraging decision-making in social insects. We compare the neuroanatomy of DA circuits related to risk-related decision making in vertebrates and insects. DA circuitry and functional mechanisms have been extensively studied in *Drosophila melanogaster*. Research on DA’s role in social insects, including honey bees and ants, remains limited, in part because their biology is not compatible with some advanced genetic tools. We consider similarities between *Drosophila* DA circuits and those of social insects and how these circuits may support behavioral adaptations and resilience to changing environmental conditions.

## 2 DA regulates individual foraging decisions

Foraging behavior is often used as a model to investigate learning processes. In these operational conditioning studies, food serves as a reward to train animals to perform a task, and negative consequences, such as shocks, act as deterrents. Animals must decide whether to engage in foraging behavior or abstain to avoid potential punishment.

Studies involving vertebrates reveal that DA levels significantly affect decisions regarding food rewards and associated risks (26, 32–36). DA release in the nucleus accumbens (NAc), part of the ventral striatum, is particularly important for decisions about whether to engage in risk-taking or risk-averse behavior (27). When the NAc is impaired, rats tend to exhibit risk-averse behavior (37). In rats, the NAc also plays a crucial role in motivation, reward, motor function, and learning (38–41).

In *Drosophila*, DA regulates foraging behavior in response to hunger and satiation through two distinct dopaminergic circuits (42). Both circuits facilitate foraging behavior regardless of hunger state (43) and converge on Kenyon cells in the mushroom body (MB), a region important for learning (44). Increased MB DAN activity causes hungry fruit flies to overcome their aversion and be more motivated to engage in risky foraging behavior (45, 46). DA mediates decisions about food choice based on nutritional value, and DANs in the MB are critical for learning the value of beneficial and harmful food components (47).

## 3 Social decision-making and risk

Many animals use social information in decisions about risk. Vertebrates that live in groups, such as insectivorous bats, starlings and bison use social information, such as visual and vocal cues from members of their group, to evaluate food resources, avoid predators and find nests (48–51). For example, scavengers such as vultures rely on social information, as they forage on patchy resources that can be difficult for an individual to discover and hunt on its own (51). In vertebrates, DA is important for social interactions (52–54), sexual behavior (55), and social hierarchy (56). However, little is known about the role of DA in vertebrate group decision-making.

Social insects, such as honey bees and ants, live in colonies that work collectively. Individuals respond to social cues, mostly olfactory (57, 58), that in the aggregate adjust the activity of the colony (59, 60). Social information influences decisions about activity outside the nest, which entails exposure to risks such as predation and environmental stressors. Individual decisions about foraging contribute to the collective regulation of foraging in social insect colonies, in which food is not directly consumed by foragers. Thus, foraging does not provide an immediate reward to the individual forager. Instead, food is brought back to the nest to be shared with the rest of the colony.

In honey bees, DA acts on neural circuits and receptors associated with both rewarding stimuli and the avoidance of risk. Increased DA is associated with increased motivation for foraging activity, and brain DA levels drop sharply, within seconds, when food is obtained (22). Predation risk, assessed as an encounter with a predator or a nestmate that experienced predation stress, reduces both foraging activity and brain DA levels (61, 62). A pharmacological increase in DA decreases the fear response, rescues foraging activity (61, 62), and decreases aggressiveness in response to aversive stimuli (63). In contrast, DA can increase during avoidance learning (64) and punishment (65).

In ants, there have been few studies of the role of DA in decisions related to risk (72, 73), although the conserved functions of biogenic amines in insects suggest that DA is involved (66). In some species, brain DA levels are highest in foragers, who must decide whether to risk exposure to hazardous conditions outside the nest (67–70). Starvation also reduces DA levels, which in turn reduces the likelihood that an ant will distribute food to its nestmates (71); however, social feeding from other ants can restore brain DA levels. DA is also involved in an ant’s decision whether to defend or retreat when facing danger (72). DA supports learning the cuticular hydrocarbon profiles of other ants (73), which are used in nestmate recognition. DA increases threatening behavior and aggression towards both other ants (74) and prey (76) while reducing affiliatory behavior toward nestmates (74). The decision whether to attack or accept another individual is associated with the risk of injury in fighting or harm if a colony is invaded.

## 4 Function of DA in foraging decision of harvester ants

In red harvester ants (*Pogonomyrmex barbatus*), DA plays a role in decisions about the risk of water loss (74). In the desert, colonies face a trade-off between water loss and foraging (75). A forager loses water to evaporation when outside the nest searching for seeds, while colonies obtain water by metabolizing the fats from the seeds they eat (76). Thus, a colony must spend water to obtain water and food. Colonies manage this tradeoff using feedback from olfactory interactions inside the nest. An outgoing forager decides to leave the nest on its next trip using simple olfactory interactions: antennal contact with returning foragers bringing in food (77–79). During antennal contact, the outgoing forager assesses the task-specific cuticular hydrocarbon profile of the returning forager as well as the odor of the food the returning forager is carrying (80–82). A forager’s decision whether to leave the nest depends on the rate of encounter with returning foragers, which relies on excitable dynamics analogous to leaky integration by neurons (83). The rate of forager return, and thus the rate of encounter with returning foragers, provides positive feedback from food availability, because higher food availability leads foragers to find food faster (84), resulting in a higher rate of forager return.

A forager’s decision whether to leave the nest to search for food also depends on the humidity the forager experienced on its last trip (85). In humid conditions, foraging tends to be high in all colonies (86). In dry conditions, colonies differ in forager decisions about whether to leave the nest on the next trip (87). Colonies show characteristic, consistent behavior across a gradient (86). At one extreme are the Risk Averse (RA) colonies, with foragers unlikely to leave the nest on the next trip in hot, dry conditions. These colonies sacrifice food intake to conserve water. At the other extreme are the Risk Tolerant (RT) colonies, where foragers continue to leave the nest on the next trip in hot, dry conditions.

Colony differences in risk aversion of foragers persist from year to year. In this long-lived species, the queen or reproductive female in the colony produces all the workers and reproductives over her 20-30-year lifetime while the workers live only a year (88). Within a colony, the foragers show similar responses to current humidity, sharing the threshold low humidity at which they will not leave the nest (89). These results, showing consistent behavior in successive cohorts of workers of a particular colony, indicate that forager decisions about the risk of low humidity are associated with heritable traits passed down from the queen and her mates. Heritable variation among colonies can be shaped by natural selection. Early in the current drought, natural selection was favoring the RA colonies that conserve water (87), but this may change as the drought intensifies and the food supply declines (75).

**Figure 1:**
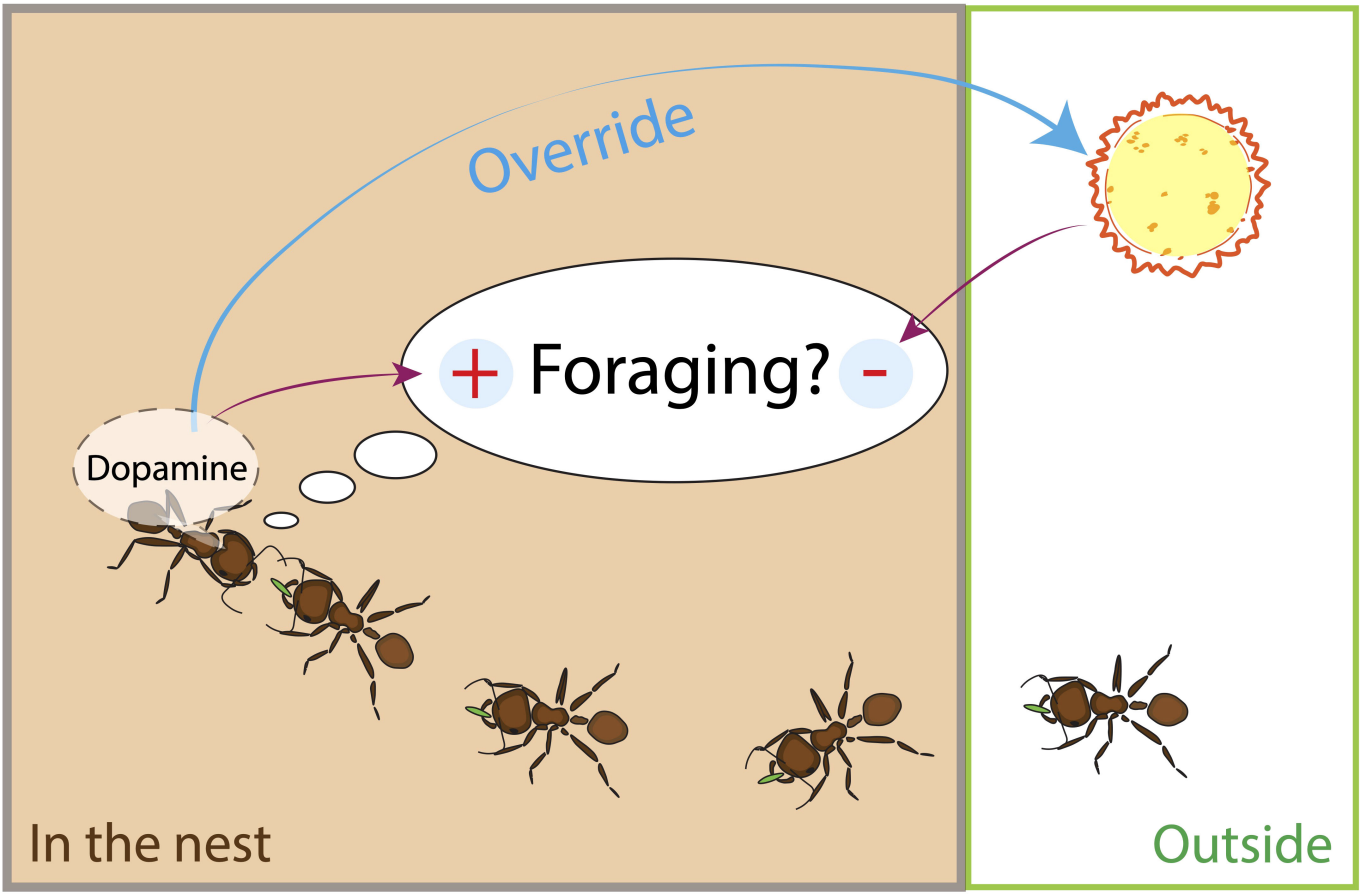
A harvester ant forager decides whether to leave the nest on another foraging trip by using its rate of antennal contact with returning foragers with food. During antennal contact, the forager assesses the odor of the cuticular hydrocarbons of the other ant, and the odor of the food it is carrying. Foragers lose water to evaporation when outside the nest. Dry conditions and desiccation experienced on previous foraging trips inhibit the decision to leave on another foraging trip. Dopamine (DA) overrides the forager’s assessment of the risk due to dry conditions.

Previous work indicates that DA may play a role in foraging decisions about the risk of water loss. First, transcriptomic analysis shows that the expression of genes related to DA metabolism is lower in RA colonies, which tend to reduce foraging in dry conditions (90). Second, pharmacological experiments demonstrate that ants fed with DA were more likely to increase their foraging trips, and the effect was most pronounced in RA colonies (74). These results suggest that DA can override risk aversion (**Figure 1**). Current research is investigating whether DA is lower in RA colonies, asking how DANs differ in the brains of foragers from RA and RT colonies, and how DA levels are influenced by current humidity conditions.

## 5 Neuroanatomy of dopaminergic neurons in relation to risk

One of the challenges in investigating the role of DA in risk-related decisions in social insects is that it is difficult to manipulate gene expression because many species do not reproduce in the lab. Other tools for mapping the anatomy and function of specific neurons in ants, such as immunohistochemistry, make it possible to compare the DA circuits in *D. melanogaster*, where genetic techniques are well-developed, with those in honey bees and ants.

**Figure 2:**
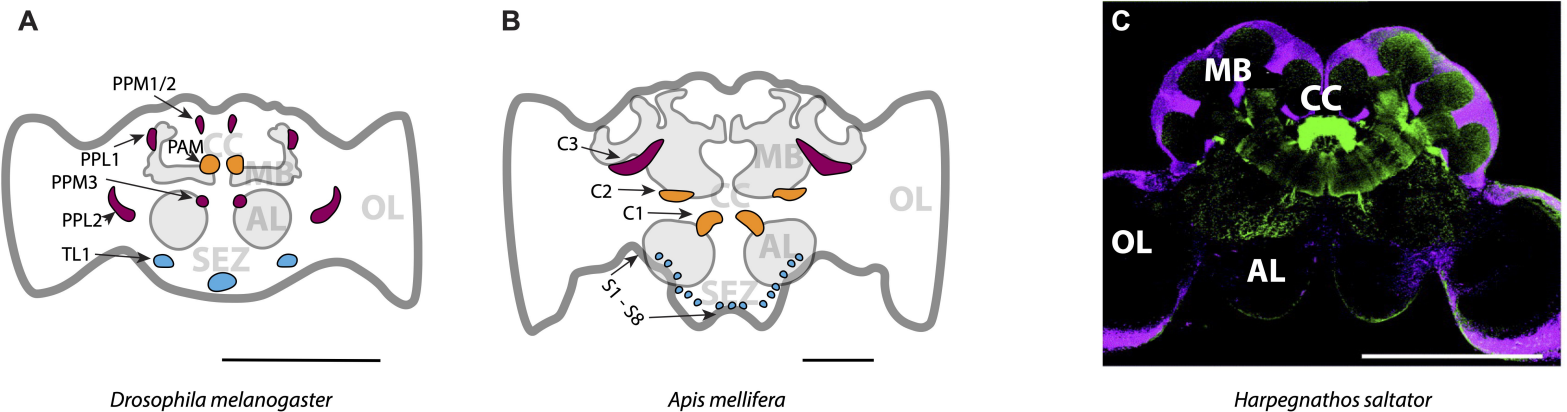
Main dopaminergic clusters mapped in **(A)** the fruit fly *Drosophila melanogaster*, **(B)** the honey bee *Apis mellifera*, and **(C)** the Indian jumping ant *Harpegnathos saltator*. Only one hemisphere of the brain is labeled. Expected homologous clusters are shown in the same color across species in **(A, B)**. Clusters are indicated with black letters and arrows, while functionally distinct brain regions are labeled in grey. In **(C)**, a confocal micrograph shows dopamine immunoreactivity in green, with brain regions visualized using propidium iodide-labeled nuclei in magenta. MB, Mushroom body; CC, central complex; AL, antennal lobe; OL, optic lobe and SEZ, subesophageal zone. Scale bars: 200 µm **(A, B)**; 500 µm **(C)**. Source: **(A, B)** adapted from Tedjakumala et al. (112), published under CC BY 4.0; **(C)** adapted with permission from Hoyer et al. (113), © 2005 Elsevier Ltd.

Mapping of DAN populations in the *Drosophila* brain reveals DA circuits that modulate learning in response to reward or punishment (**Figure 2A**). The *Drosophila* brain contains 130–140 DANs distributed across 13 distinct clusters in each hemisphere (91–93). Heterogeneous populations of DANs work together to influence risk-based decisions (94, 95). DAN clusters in the anterior medial part of the brain (PAM), which project to the MB, are required for reward learning (96–98) and resolving conflict between aversive and rewarding stimuli (45, 99, 100). Short-term memory formation associated with aversive and reward-seeking behavior is mediated by PAM DANs through MB compartments β’2 and γ4, while the signals necessary for long-term memory formation regarding reward, such as the nutritional value of sugar, are relayed by DANs projecting to MB compartments γ5, β1, β2, α1, and γ1pedc (97, 101–105). Certain clusters within the MB are critical for promoting food search efforts in response to food odor, while inactivation of specific DAN subsets within the protocerebral posterior lateral (PPL) region, clusters PPL1 and PPL2, significantly impair odor-tracking behavior (97). A particular group of DANs in one of the protocerebral posterior medial (PPM) clusters (PPM2), is connected to wedge neurons in the central complex and influences state-dependent decisions to consume protein-rich food (106), while the PPM3 cluster is responsible for food-seeking in fed flies (42).

MB DANs control temperature preference and mediate avoidance responses to both low and high temperatures (107, 108). Gene expression related to DA synthesis and release is regulated in MB DAN clusters in response to temperature (109). DAN clusters in the MB (PPL1-α3/α’3, PPL1-α2α’2, PPL1-γ2α’1, and PPL1-γ1pedc) respond when the temperature is lower than the optimum (110), and the MB clusters PAM-β’2 and PAM-β2 are also involved in avoiding low temperatures (111).

DAN clusters identified in honey bee brains have shown patterns similar to those in *Drosophila*. Several clusters in the MB have been identified as homologous to those in *Drosophila* that support foraging decisions and sense environmental signals (**Figure 2B**). In honey bees, the C1 and C2 clusters may be homologous to the *Drosophila* PAM cluster, used in foraging (112). Neurons in the C3 cluster may be the homologues of PPL1, PPL2 and PPM3, which are important for odor-tracking (112).

In ants, DAN clusters have dendritic and axonal projections in most regions of the brain (**Figure 2C**). For example, in the brains of Indian jumping ants, *Harpegnathos saltator*, there are clusters in the MB and antennal lobes, the primary olfactory processing regions, with scattered cell bodies in the optic lobe, the primary visual processing region, and the subesophageal zone, a region involved in feeding behavior and learning (113). However, it is not yet known whether any of these neurons are homologous to the DANs in *Drosophila*.

## 6 DA receptors

DA receptors are similar in vertebrates and insects. In vertebrates, there are two classes of DA G-protein-coupled receptors (GPCRs): D1 and D2. The D1 class, which includes two types of DA receptors, D1 and D5, increases intracellular cAMP levels, leading to excitatory actions in the brain that facilitate neurotransmitter release and trigger behavioral responses (114). The receptors in the D1 class are involved in memory, attention, motivation, and movement (115). In contrast, the D2 class, which includes the other three types, D2, D3, and D4, mediates the reduction of cAMP, often resulting in inhibitory effects that modulate neurotransmitter release (116). D2 class receptors are involved in mood and motor regulation (117). D1, D2, and D3 types are the most abundant receptors in the central nervous system (18).

Both D1 and D2 type receptors are important for making foraging decisions in rats (33). After treatment with a D1 or D2 antagonist, rats showed a greater tendency to choose a smaller but certain reward. Conversely, administration of a DA agonist increased the likelihood of selecting a large but risky reward option and mitigated the effects of both antagonists. Treatment with a D3 receptor antagonist did not significantly affect choice behavior.

*Drosophila* have four types of DA receptors, which are widely expressed in the brain. These receptors include Dop1R1 and Dop1R2, both of which are homologous to vertebrate D1 class receptors and are implicated in motivation-related behavior such as arousal, drug reward, and learning and memory (42, 118, 119). Activation of Dop1R1 in the MB increases the activity of MB output neurons and encodes long-term memory (120). Mechanistic studies on aversive memory suggest that as DA levels decline over time, Dop1R2 acts within the same neurons as Dop1R1 to facilitate forgetting (121). Additionally, Dop1R2 receptor signaling in α/β Kenyon cells (KCs) has been implicated in modulating motivated search behavior (46). Dop2R, analogous to vertebrate D2 class receptors, seems to play an opposing role to D1-like receptors (42). Dop2R activation in GABAergic anterior paired lateral (APL) neurons, which innervate the MB, plays a critical role in aversive conditioning by restraining GABAergic inhibition (122). The fourth receptor, DopEcR, is unique to *D. melanogaster*. When activated by DA it leads to neuronal excitation (123), though its role in foraging and reward-seeking behavior is not yet understood.

As in *Drosophila*, honey bees have both the D1 class-like and D2 class-like receptors (124): AmDop1 and AmDop2 (125) are homologues of DopR1 and DopR2 in *Drosophila*. AmDop2 regulates worker movement (126), but it is not known whether these DA receptors regulate foraging or reward learning behavior. AmDop3, a homolog of Dop2R and D2 class receptors in vertebrates, has been confirmed to be a third DA receptor in honey bee because its expression pattern in the honey bee brain is different from that of either Amdop1or Amdop2 (127).

In ants, no DA receptors have been identified or categorized yet. However, DA receptor antagonists, such as flupentixol, a general D1- and D2-like receptor antagonist (128), have been used to study the function of DA in foraging preferences. *Lasius niger* workers treated with flupentixol initially learned an odor linked to a reward but failed to retrieve this memory 24 hours later (73). This suggests that DA in ants acts on similar DA receptors as in other insects and is essential for long-term memory in ants.

To date, no mapping of DANs or DA receptors has been conducted in harvester ant brains. Future research will focus on identifying the types and distribution of DA receptors in ants and mapping their dopaminergic circuitry. By comparing these findings with existing data from *Drosophila* and honey bees, we can gain valuable insight into the evolutionary conservation and functional roles of DA in regulating foraging decisions in social insects. Understanding how dopaminergic pathways influence foraging behavior in ants will provide a broader perspective on the neuromodulatory mechanisms underlying collective decision-making in eusocial species.

## 7 Conclusions

DA plays a crucial role in risk-based decision-making across diverse animal species and influences individual decisions that lead to the collective regulation of foraging activity in social insect colonies. Research in vertebrates and *Drosophila* has revealed well-defined dopaminergic circuits that regulate reward perception, motivation, and risk assessment.

Integrating pharmacological manipulations and behavioral analyses with neuroanatomical comparisons of dopaminergic cell expression and receptor distributions across *Drosophila*, honey bees, and ants will show whether neural pathways involved in decision-making are evolutionarily conserved. This can elucidate the mechanisms of decision-making in collective behavior, and predict the possibilities for the adaptation of social organisms to changing environmental conditions.

## References

[1] Yoshimura J Clark CW. Individual adaptations in stochastic environments. Evol Ecol. (1991) 5:173–92. doi: 10.1007/BF02270833

[2] Lima SL. Stress and decision making under the risk of predation: recent developments from behavioral, reproductive, and ecological perspectives. Adv Study Behav. (1998) 27:215–90. doi: 10.1016/S0065-3454(08)60366-6

[3] Polis GA Myers CA Holt RD. The ecology and evolution of intraguild predation: potential competitors that eat each other. Annu Rev Ecol Syst. (1989) 20:297–330. doi: 10.1146/annurev.es.20.110189.001501

[4] McNamara JM. The starvation-predation trade-off and some behavioural and ecological consequences. In: Behavioural mechanisms of food selection. Berlin Heidelberg: Springer (1990). p. 39–59.

[5] Caraco T Blanckenhorn WU Gregory GM Newman JA Recer GM Zwicker SM. Risk-sensitivity: ambient temperature affects foraging choice. Anim Behav. (1990) 39:338–45. doi: 10.1016/S0003-3472(05)80879-6

[6] Stahlschmidt ZR Brashears J DeNardo DF. The role of temperature and humidity in python nest site selection. Anim Behav. (2011) 81:1077–81. doi: 10.1016/j.anbehav.2011.02.024

[7] Draper AM Weissburg MJ. Impacts of global warming and elevated CO2 on sensory behavior in predator-prey interactions: A review and synthesis. Front Ecol Evol. (2019) 7:72. doi: 10.3389/fevo.2019.00072

[8] Beever EA Hall LE Varner J Loosen AE Dunham JB Gahl MK. Behavioral flexibility as a mechanism for coping with climate change. Front Ecol Environ. (2017) 15:299–308.

[9] Bergstrom DM Wienecke BC Van den Hoff J Hughes L Lindenmayer DB Ainsworth TD. Combating ecosystem collapse from the tropics to the Antarctic. Glob Chang Biol. (2021) 27:1692–703. doi: 10.1002/fee.1502 33629799

[10] Both C. Food availability, mistiming, and climatic change. In Møller AP Fiedler W Berthold PEds. Effects of climate change on birds. OUP Oxford (2010) 129–1482.

[11] Traill LW Lim MLM Sodhi NS Bradshaw CJA. Mechanisms driving change: altered species interactions and ecosystem function through global warming. J Anim Ecol. (2010) 79:937–47. doi: 10.1111/j.1365-2656.2010.01695.x 20487086

[12] Rogers RD. The roles of dopamine and serotonin in decision making: evidence from pharmacological experiments in humans. Neuropsychopharmacology. (2011) 36:114–32. doi: 10.1038/npp.2010.165 PMC3055502 20881944

[13] Doya K. Modulators of decision making. Nat Neurosci. (2008) 11:410–6. doi: 10.1038/nn2077 18368048

[14] Garris PA Rebec GV. Modeling fast dopamine neurotransmission in the nucleus accumbens during behavior. Behav Brain Res. (2002) 137:47–63. doi: 10.1016/S0166-4328(02)00284-X 12445715

[15] Westerink RHS. Targeting exocytosis: ins and outs of the modulation of quantal dopamine release. CNS Neurol Disord Targets. (2006) 5:57–77. 10.2174/187152706784111597 16613554

[16] Grace AA Floresco SB Goto Y Lodge DJ. Regulation of firing of dopaminergic neurons and control of goal-directed behaviors. Trends Neurosci. (2007) 30:220–7. doi: 10.2174/187152706775535632 17400299

[17] Liu C Kaeser PS. Mechanisms and regulation of dopamine release. Curr Opin Neurobiol. (2019) 57:46–53. doi: 10.1016/j.conb.2019.01.001 30769276 PMC6629510

[18] Mishra A Singh S Shukla S. Physiological and functional basis of dopamine receptors and their role in neurogenesis: possible implication for Parkinson’s disease. J Exp Neurosci. (2018) 12. doi: 10.1177/1179069518779829 PMC5985548 29899667

[19] Bromberg-Martin ES Hikosaka O. Midbrain dopamine neurons signal preference for advance information about upcoming rewards. Neuron. (2009) 63:119–26. doi: 10.1016/j.neuron.2009.06.009 PMC2723053 19607797

[20] Nestler EJ Carlezon WA Jr. The mesolimbic dopamine reward circuit in depression. Biol Psychiatry. (2006) 59:1151–9. doi: 10.1016/j.biopsych.2005.09.018 16566899

[21] Roesch MR Calu DJ Schoenbaum G. Dopamine neurons encode the better option in rats deciding between differently delayed or sized rewards. Nat Neurosci. (2007) 10:1615–24. doi: 10.1038/nn2013 PMC2562672 18026098

[22] Huang J Zhang Z Feng W Zhao Y Aldanondo A de Brito Sanchez MG. Food wanting is mediated by transient activation of dopaminergic signaling in the honey bee brain. Science. (2022) 376:508–12. doi: 10.1126/science.abn9920 35482873

[23] Rajagopalan AE Darshan R Hibbard KL Fitzgerald JE Turner GC. Reward expectations direct learning and drive operant matching in Drosophila. Proc Natl Acad Sci U S A. (2023) 120:1–12. doi: 10.1073/pnas.2221415120 PMC10523640 37733736

[24] Bromberg-Martin ES Matsumoto M Hikosaka O. Dopamine in motivational control: rewarding, aversive, and alerting. Neuron. (2010) 68:815–34. doi: 10.1016/j.neuron.2010.11.022 PMC3032992 21144997

[25] Verharen JPH Adan RAH Vanderschuren LJMJ. How reward and aversion shape motivation and decision making: A computational account. Neuroscientist. (2020) 26:87–99. doi: 10.1177/1073858419834517 30866712

[26] Freels TG Gabriel DBK Lester DB Simon NW. Risky decision-making predicts dopamine release dynamics in nucleus accumbens shell. Neuropsychopharmacology. (2020) 45:266–75. doi: 10.1038/s41386-019-0527-0 PMC6901435 31546248

[27] Sugam JA Day JJ Wightman RM Carelli RM. Phasic nucleus accumbens dopamine encodes risk-based decision-making behavior. Biol Psychiatry. (2012) 71:199–205. https://www.sciencedirect.com/science/article/pii/S0006322311009498 (Accessed April 9, 2025). 22055017 10.1016/j.biopsych.2011.09.029 PMC3253943

[28] Young JW van Enkhuizen J Winstanley CA Geyer MA. Increased risk-taking behavior in dopamine transporter knockdown mice: further support for a mouse model of mania. J Psychopharmacol. (2011) 25:934–43. doi: 10.1177/0269881111400646 PMC3568506 21421642

[29] Norbury A Manohar S Rogers RD Husain M. Dopamine modulates risk-taking as a function of baseline sensation-seeking trait. J Neurosci. (2013) 33:12982–6. doi: 10.1523/JNEUROSCI.5587-12.2013 PMC3735881 23926253

[30] Clark CA Dagher A. The role of dopamine in risk taking: a specific look at Parkinson’s disease and gambling. Front Behav Neurosci. (2014) 8:196. doi: 10.3389/fnbeh.2014.00196 24910600 PMC4038955

[31] Kohno M Ghahremani DG Morales AM Robertson CL Ishibashi K Morgan AT. Risk-taking behavior: dopamine D2/D3 receptors, feedback, and frontolimbic activity. Cereb Cortex. (2015) 25:236–45. doi: 10.1093/cercor/bht218 PMC4259280 23966584

[32] St Onge JR Floresco SB. Dopaminergic modulation of risk-based decision making. Neuropsychopharmacology. (2009) 34:681–97. doi: 10.1038/npp.2008.121 18668030

[33] Bardgett ME Depenbrock M Downs N Points M Green L. Dopamine modulates effort-based decision making in rats. Behav Neurosci. (2009) 123:242–51. doi: 10.1037/a0014625 PMC2791340 19331447

[34] Cai X Liu C Tsutsui-Kimura I Lee JH Guo C Banerjee A. Dopamine dynamics are dispensable for movement but promote reward responses. Nature. (2024) 635:1–9. doi: 10.1038/s41586-024-08038-z PMC11718420 39415006

[35] Messias JPM Paula JR Grutter AS Bshary R Soares MC. Dopamine disruption increases negotiation for cooperative interactions in a fish. Sci Rep. (2016) 6:2–9. doi: 10.1038/srep20817 26853241 PMC4745044

[36] Alikaya A Rack-Wildner M Stauffer WR. Reward and value coding by dopamine neurons in non-human primates. J Neural Transm. (2018) 125:565–74. doi: 10.1007/s00702-017-1793-9 PMC5847197 29076112

[37] Stopper CM Floresco SB. Contributions of the nucleus accumbens and its subregions to different aspects of risk-based decision making. Cognit Affect Behav Neurosci. (2011) 11:97–112. doi: 10.3758/s13415-010-0015-9 21264647

[38] Hikida T Morita M Macpherson T. Neural mechanisms of the nucleus accumbens circuit in reward and aversive learning. Neurosci Res. (2016) 108:1–5. doi: 10.1016/j.neures.2016.01.004 26827817

[39] Carlezon WA Jr. Thomas MJ. Biological substrates of reward and aversion: a nucleus accumbens activity hypothesis. Neuropharmacology. (2009) 56:122–32. doi: 10.1016/j.neuropharm.2008.06.075 PMC2635333 18675281

[40] Yawata S Yamaguchi T Danjo T Hikida T Nakanishi S. Pathway-specific control of reward learning and its flexibility via selective dopamine receptors in the nucleus accumbens. Proc Natl Acad Sci. (2012) 109:12764–9. doi: 10.1073/pnas.1210797109 PMC3412032 22802650

[41] Sawada M Kato K Kunieda T Mikuni N Miyamoto S Onoe H. Function of the nucleus accumbens in motor control during recovery after spinal cord injury. Science. (2015) 350:98–101. doi: 10.1126/science.aab3825 26430122

[42] Landayan D Feldman DS Wolf FW. Satiation state-dependent dopaminergic control of foraging in Drosophila. Sci Rep. (2018) 8:1–9. doi: 10.1038/s41598-018-24217-1 29636522 PMC5893590

[43] Tsao CH Chen CC Lin CH Yang HY Lin S. *Drosophila* mushroom bodies integrate hunger and satiety signals to control innate food-seeking behavior. Elife. (2018) 7:1–35. doi: 10.7554/eLife.35264 PMC5910021 29547121

[44] Davis RL. Mushroom bodies and *Drosophila* learning. Neuron. (1993) 11:1–14. doi: 10.1016/0896-6273(93)90266-T 8338661

[45] Lewis LPC Siju KP Aso Y Friedrich AB Bulteel AJB Rubin GM. A higher brain circuit for immediate integration of conflicting sensory information in Drosophila. Curr Biol. (2015) 25:2203–14. doi: 10.1016/j.cub.2015.07.015 26299514

[46] Sayin S De Backer J-F Siju KP Wosniack ME Lewis LP Frisch L-M. A neural circuit arbitrates between persistence and withdrawal in hungry Drosophila. Neuron. (2019) 104:544–58. doi: 10.1016/j.neuron.2019.07.028 PMC6839618 31471123

[47] Das G Lin S Waddell S. Remembering components of food in Drosophila. Front Integr Neurosci. (2016) 10:1–8. doi: 10.3389/fnint.2016.00004 26924969 PMC4759284

[48] Dechmann DKN Heucke SL Giuggioli L Safi K Voigt CC Wikelski M. Experimental evidence for group hunting via eavesdropping in echolocating bats. Proc R Soc B Biol Sci. (2009) 276:2721–8. doi: 10.1098/rspb.2009.0473 PMC2839959 19419986

[49] Powell GVN. Experimental analysis of the social value of flocking by starlings (*Sturnus vulgaris*) in relation to predation and foraging. Anim Behav. (1974) 22:501–5. doi: 10.1016/S0003-3472(74)80049-7

[50] Sigaud M Merkle JA Cherry SG Fryxell JM Berdahl A Fortin D. Collective decision-making promotes fitness loss in a fusion-fission society. Ecol Lett. (2017) 20:33–40. doi: 10.1111/ele.2017.20.issue-1 27873440

[51] Gil MA Hein AM Spiegel O Baskett ML Sih A. Social information links individual behavior to population and community dynamics. Trends Ecol Evol. (2018) 33:535–48. doi: 10.1016/j.tree.2018.04.010 29748042

[52] Cutando L Puighermanal E Castell L Tarot P Belle M Bertaso F. Cerebellar dopamine D2 receptors regulate social behaviors. Nat Neurosci. (2022) 25:900–11. doi: 10.1038/s41593-022-01092-8 35710984

[53] Bergey CM Phillips-Conroy JE Disotell TR Jolly CJ. Dopamine pathway is highly diverged in primate species that differ markedly in social behavior. Proc Natl Acad Sci. (2016) 113:6178–81. doi: 10.1073/pnas.1525530113 PMC4896724 27140612

[54] Yamaguchi Y Atsumi T Poirot R Lee Y-A Kato A Goto Y. Dopamine-dependent visual attention preference to social stimuli in nonhuman primates. Psychopharmacol (Berl). (2017) 234:1113–20. doi: 10.1007/s00213-017-4544-6 PMC5352745 28154891

[55] Graham MD Pfaus JG. Differential effects of dopamine antagonists infused to the medial preoptic area on the sexual behavior of female rats primed with estrogen and progesterone. Pharmacol Biochem Behav. (2012) 102:532–9. doi: 10.1016/j.pbb.2012.06.020 22750065

[56] Yamaguchi Y Lee Y-A Kato A Goto Y. The roles of dopamine D1 receptor on the social hierarchy of rodents and nonhuman primates. Int J Neuropsychopharmacol. (2017) 20:324–35. doi: 10.1093/ijnp/pyw106 PMC5409125 27927739

[57] Richard F-J Hunt JH. Intracolony chemical communication in social insects. Insectes Soc. (2013) 60:275–91. doi: 10.1007/s00040-013-0306-6

[58] Barrows EM Bell WJ Michener CD. Individual odor differences and their social functions in insects. Proc Natl Acad Sci. (1975) 72:2824–8. doi: 10.1073/pnas.72.7.2824 PMC432864 1058498

[59] Ali MF Morgan ED. Chemical communication in insect communities: a guide to insect pheromones with special emphasis on social insects. Biol Rev. (1990) 65:227–47. doi: 10.1111/j.1469-185X.1990.tb01425.x

[60] Kannan K Galizia CG Nouvian M. Olfactory strategies in the defensive behaviour of insects. Insects. (2022) 13:470. doi: 10.3390/insects13050470 35621804 PMC9145661

[61] Gu G Wang Z Lin T Wang S Li J Dong S. Bee fear responses are mediated by dopamine and influence cognition. J Anim Ecol. (2024) 4):1–13. doi: 10.1111/1365-2656.14224 39562840

[62] Dong S Gu G Lin T Wang Z Li J Tan K. An inhibitory signal associated with danger reduces honeybee dopamine levels. Curr Biol. (2023) 33:2081–2087.e4. doi: 10.1016/j.cub.2023.03.072 37059097

[63] Nouvian M Mandal S Jamme C Claudianos C D’Ettorre P Reinhard J. Cooperative defence operates by social modulation of biogenic amine levels in the honey bee brain. Proc R Soc B Biol Sci. (1871) 2018:285. doi: 10.1098/rspb.2017.2653 PMC5805953 29367399

[64] Agarwal M Guzmán M Morales-Matos C Del Valle Díaz RA Abramson CI Giray T. Dopamine and octopamine influence avoidance learning of honey bees in a place preference assay. PloS One. (2011) 6:1–9. doi: 10.1371/journal.pone.0025371 PMC3184138 21980435

[65] Jarriault D Fuller J Hyland BI Mercer AR. Dopamine release in mushroom bodies of the honey bee (*Apis mellifera* L.) in response to aversive stimulation. Sci Rep. (2018) 8:1–12. doi: 10.1038/s41598-018-34460-1 30389979 PMC6214997

[66] Kamhi JF Arganda S Moreau CS Traniello JFA. Origins of aminergic regulation of behavior in complex insect social systems. Front Syst Neurosci. (2017) 11:1–9. doi: 10.3389/fnsys.2017.00074 29066958 PMC5641352

[67] Okada Y Sasaki K Miyazaki S Shimoji H Tsuji K Miura T. Social dominance and reproductive differentiation mediated by dopaminergic signaling in a queenless ant. J Exp Biol. (2015) 218:1091–8. doi: 10.1242/jeb.118414 25687437

[68] Seid MA Harris KM Traniello JFA. Age-related changes in the number and structure of synapses in the lip region of the mushroom bodies in the ant Pheidole dentata. J Comp Neurol. (2005) 488:269–77. doi: 10.1002/(ISSN)1096-9861 15952165

[69] Seid MA Traniello JFA. Age-related repertoire expansion and division of labor in *Pheidole dentata* (Hymenoptera: Formicidae): a new perspective on temporal polyethism and behavioral plasticity in ants. Behav Ecol Sociobiol. (2006) 60:631–44. doi: 10.1007/s00265-006-0207-z

[70] Smith AR Muscedere ML Seid MA Traniello JFA Hughes WOH. Biogenic amines are associated with worker task but not patriline in the leaf-cutting ant Acromyrmex eChinatior. J Comp Physiol A. (2013) 199:1117–27. doi: 10.1007/s00359-013-0854-2 24072064

[71] Wheeler WM Bailey IW. The feeding habits of *Pseudomyrmine* and other ants. Trans Am Philos Soc. (1920) 22:235–79. http://www.jstor.org/stable/1005485.

[72] Aonuma H. Serotonergic control in initiating defensive responses to unexpected tactile stimuli in the trap-jaw ant *Odontomachus kuroiwae* . J Exp Biol. (2020) 223(19):jeb228874. doi: 10.1242/jeb.228874 (Accessed April 9, 2025). 32895325

[73] Wissink M Nehring V. Appetitive olfactory learning suffers in ants when octopamine or dopamine receptors are blocked. J Exp Biol. (2021) 224:jeb242732. doi: 10.1242/jeb.242732 34357377

[74] Friedman DA Pilko A Skowronska-Krawczyk D Krasinska K Parker JW Hirsh J. The role of dopamine in the collective regulation of foraging in harvester ants. iScience. (2018) 8:283–94. doi: 10.1016/j.isci.2018.09.001 PMC6205345 30270022

[75] Sundaram M Steiner E Gordon DM. Rainfall, neighbors, and foraging: The dynamics of a population of red harvester ant colonies 1988–2019. Ecol Monogr. (2022) 92:1–29. doi: 10.1002/ecm.v92.2

[76] Lighton JRB Feener DH Jr. Water-loss rate and cuticular permeability in foragers of the desert ant Pogonomyrmex rugosus. Physiol Zool. (1989) 62:1232–56. doi: 10.1086/physzool.62.6.30156211

[77] Gordon DM Guetz A Greene MJ Holmes S. Colony variation in the collective regulation of foraging by harvester ants. Behav Ecol. (2011) 22:429–35. doi: 10.1093/beheco/arq218 PMC3071749 22479133

[78] Prabhakar B Dektar KN Gordon DM. The regulation of ant colony foraging activity without spatial information. PloS Comput Biol. (2012) 8:e1002670. doi: 10.1371/journal.pcbi.1002670 22927811 PMC3426560

[79] Schafer RJ Holmes S Gordon DM. Forager activation and food availability in harvester ants. Anim Behav. (2006) 71:815–22. doi: 10.1016/j.anbehav.2005.05.024 PMC3767282 24031094

[80] Greene MJ Gordon DM. Cuticular hydrocarbons inform task decisions. Nature. (2003) 423:32. doi: 10.1038/423032a 12721617

[81] Wagner D Tissot M Cuevas W Gordon DM. Harvester ants utilize cuticular hydrocarbons in nestmate recognition. J Chem Ecol. (2000) 26:2245–57. doi: 10.1023/A:1005529224856

[82] Greene MJ Pinter-Wollman N Gordon DM. Interactions with combined chemical cues inform harvester ant foragers’ Decisions to leave the nest in search of food. PloS One. (2013) 8:1–8. doi: 10.1371/journal.pone.0052219 PMC3540075 23308106

[83] Davidson JD Arauco-Aliaga RP Crow S Gordon DM Goldman MS. Effect of interactions between harvester ants on forager decisions. Front Ecol Evol. (2016) 4:115. doi: 10.3389/fevo.2016.00115 28758093 PMC5531068

[84] Beverly BD McLendon H Nacu S Holmes S Gordon DM. How site fidelity leads to individual differences in the foraging activity of harvester ants. Behav Ecol. (2009) 20:633–8. doi: 10.1093/beheco/arp041

[85] Pagliara R Gordon DM Leonard NE. Regulation of harvester ant foraging as a closed-loop excitable system. PloS Comput Biol. (2018) 14:1–25. doi: 10.1371/journal.pcbi.1006200 PMC6294393 30513076

[86] Gordon DM Steiner E Das B Walker NS. Harvester ant colonies differ in collective behavioural plasticity to regulate water loss. R Soc Open Sci. (2023) 10:230726. doi: 10.1098/rsos.230726 37736532 PMC10509591

[87] Gordon DM. The rewards of restraint in the collective regulation of foraging by harvester ant colonies. Nature. (2013) 498:91–3. doi: 10.1038/nature12137 23676676

[88] Gordon DM Hölldobler B. Worker longevity in harvester ants (*Pogonomyrmex*). Psyche A J Entomol. (1987) 94:341–6. doi: 10.1155/psyc.v94.3-4

[89] Nova N Pagliara R Gordon DM. Individual variation does not regulate foraging response to humidity in harvester ant colonies. Front Ecol Evol. (2022) 9:756204. doi: 10.3389/fevo.2021.756204

[90] Friedman DA York RA Hilliard AT Gordon DM. Gene expression variation in the brains of harvester ant foragers is associated with collective behavior. Commun Biol. (2020) 3:100. doi: 10.1038/s42003-020-0813-8 32139795 PMC7057964

[91] Budnik V White K. Catecholamine-containing neurons in *Drosophila melanogaster*: Distribution and development. J Comp Neurol. (1988) 268:400–13. doi: 10.1002/cne.902680309 3129458

[92] Mao Z Davis RL. Eight different types of dopaminergic neurons innervate the *Drosophila* mushroom body neuropil: Anatomical and physiological heterogeneity. Front Neural Circuits. (2009) 3:1–17. doi: 10.3389/neuro.04.005.2009 19597562 PMC2708966

[93] Nässel DR Elekes K. Aminergic neurons in the brain of blowflies and *Drosophila:* dopamine-and tyrosine hydroxylase-immunoreactive neurons and their relationship with putative histaminergic neurons. Cell Tissue Res. (1992) 267:147–67. doi: 10.1007/BF00318701 1346506

[94] Schultz W Dayan P Montague PR. A neural substrate of prediction and reward. Science. (1997) 275:1593–9. doi: 10.1126/science.275.5306.1593 9054347

[95] Watabe-Uchida M Uchida N. Multiple dopamine systems: weal and woe of dopamine. In: Cold spring harbor symposia on quantitative biology. Cold Spring Harbor Laboratory Press (2018). 83:83–95. 30787046 10.1101/sqb.2018.83.037648

[96] Liu C Plaa̧ais PY Yamagata N Pfeiffer BD Aso Y Friedrich AB. A subset of dopamine neurons signals reward for odour memory in *Drosophila* . Nature. (2012) 488:512–6. doi: 10.1038/nature11304 22810589

[97] Siju KP De Backer JF Grunwald Kadow IC. Dopamine modulation of sensory processing and adaptive behavior in flies. Cell Tissue Res. (2021) 383:207–25. doi: 10.1007/s00441-020-03371-x PMC7873103 33515291

[98] Zhang K Guo JZ Peng Y Xi W Guo A. Dopamine-mushroom body circuit regulates saliency-based decision-making in Drosophila. Science. (2007) 316:1901–4. doi: 10.1126/science.1137357 17600217

[99] Bräcker LB Siju KP Varela N Aso Y Zhang M Hein I. Essential role of the mushroom body in context-dependent CO2 avoidance in Drosophila. Curr Biol. (2013) 23:1228–34. doi: 10.1016/j.cub.2013.05.029 23770186

[100] Suh GSB de Leon SB-T Tanimoto H Fiala A Benzer S Anderson DJ. Light activation of an innate olfactory avoidance response in Drosophila. Curr Biol. (2007) 17:905–8. doi: 10.1016/j.cub.2007.04.046 17493811

[101] Huetteroth W Perisse E Lin S Klappenbach M Burke C Waddell S. Sweet taste and nutrient value subdivide rewarding dopaminergic neurons in Drosophila. Curr Biol. (2015) 25:751–8. doi: 10.1016/j.cub.2015.01.036 PMC4372253 25728694

[102] Musso P-Y Tchenio P Preat T. Delayed dopamine signaling of energy level builds appetitive long-term memory in Drosophila. Cell Rep. (2015) 10:1023–31. doi: 10.1016/j.celrep.2015.01.036 25704807

[103] Pavlowsky A Schor J Placais P-Y Preat T. A GABAergic feedback shapes dopaminergic input on the *Drosophila* mushroom body to promote appetitive long-term memory. Curr Biol. (2018) 28:1783–93. doi: 10.1016/j.cub.2018.04.040 PMC5988562 29779874

[104] Placais P-Y de Tredern É Scheunemann L Trannoy S Goguel V Han K-A. Upregulated energy metabolism in the *Drosophila* mushroom body is the trigger for long-term memory. Nat Commun. (2017) 8:15510. doi: 10.1038/ncomms15510 28580949 PMC5465319

[105] Yamagata N Ichinose T Aso Y Plaçais P-Y Friedrich AB Sima RJ. Distinct dopamine neurons mediate reward signals for short-and long-term memories. Proc Natl Acad Sci. (2015) 112:578–83. doi: 10.1073/pnas.1421930112 PMC4299218 25548178

[106] Liu Q Tabuchi M Liu S Kodama L Horiuchi W Daniels J. Branch-specific plasticity ofa bifunctional dopamine circuit encodes protein hunger. Science. (2017) 539:534–9. doi: 10.1126/science.aal3245 PMC5513152 28473588

[107] Bang S Hyun S Hong ST Kang J Jeong K Park JJ. Dopamine signalling in mushroom bodies regulates temperature-preference behaviour in *Drosophila* . PloS Genet. (2011) 7:e1001346. doi: 10.1371/journal.pgen.1001346 21455291 PMC3063753

[108] Galili DS Dylla KV Lüdke A Friedrich AB Yamagata N Wong JYH. Converging circuits mediate temperature and shock aversive olfactory conditioning in *Drosophila* . Curr Biol. (2014) 24:1712–22. doi: 10.1016/j.cub.2014.06.062 25042591

[109] Jakšić AM Karner J Nolte V Hsu SK Barghi N Mallard F. Neuronal function and dopamine signaling evolve at high temperature in *Drosophila* . Mol Biol Evol. (2020) 37:2630–40. doi: 10.1093/molbev/msaa116 32402077

[110] Tomchik SM. Dopaminergic neurons encode a distributed, asymmetric representation of temperature in *Drosophila* . J Neurosci. (2013) 33:2166–76. doi: 10.1523/JNEUROSCI.3933-12.2013 PMC3711641 23365252

[111] Shih H-W Wu C-L Chang S-W Liu T-H Sih-Yu Lai J Fu T-F. Parallel circuits control temperature preference in *Drosophila* during ageing. Nat Commun. (2015) 6:7775. doi: 10.1038/ncomms8775 26178754 PMC4518306

[112] Tedjakumala SR Rouquette J Boizeau ML Mesce KA Hotier L Massou I. A tyrosine-hydroxylase characterization of dopaminergic neurons in the honey bee brain. Front Syst Neurosci. (2017) 11:1–17. doi: 10.3389/fnsys.2017.00047 28740466 PMC5502285

[113] Hoyer SC Liebig J Rössler W. Biogenic amines in the ponerine ant *Harpegnathos saltator*: Serotonin and dopamine immunoreactivity in the brain. Arthropod Struct Dev. (2005) 34:429–40. doi: 10.1016/j.asd.2005.03.003

[114] Maurice N Tkatch T Meisler M Sprunger LK Surmeier DJ. D1/D5 dopamine receptor activation differentially modulates rapidly inactivating and persistent sodium currents in prefrontal cortex pyramidal neurons. J Neurosci. (2001) 21:2268–77. doi: 10.1523/JNEUROSCI.21-07-02268.2001 PMC6762404 11264302

[115] Vallone D Picetti R Borrelli E. Structure and function of dopamine receptors. Neurosci Biobehav Rev. (2000) 24:125–32. doi: 10.1016/S0149-7634(99)00063-9 10654668

[116] Ford CP. The role of D2-autoreceptors in regulating dopamine neuron activity and transmission. Neuroscience. (2014) 282:13–22. doi: 10.1016/j.neuroscience.2014.01.025 24463000 PMC4108583

[117] Ayano G. Dopamine: receptors, functions, synthesis, pathways, locations and mental disorders: review of literatures. J Ment Disord Treat. (2016) 2:2. doi: 10.4172/2471-271X.1000120

[118] Kim Y-C Lee H-G Han K-A. D1 dopamine receptor dDA1 is required in the mushroom body neurons for aversive and appetitive learning in *Drosophila* . J Neurosci. (2007) 27:7640–7. doi: 10.1523/JNEUROSCI.1167-07.2007 PMC6672866 17634358

[119] Lebestky T Chang J-SC Dankert H Zelnik L Kim Y-C Han K-A. Two different forms of arousal in *Drosophila* are oppositely regulated by the dopamine D1 receptor ortholog DopR via distinct neural circuits. Neuron. (2009) 64:522–36. doi: 10.1016/j.neuron.2009.09.031 PMC2908595 19945394

[120] Kaun KR Rothenfluh A. Dopaminergic rules of engagement for memory in *Drosophila* . Curr Opin Neurobiol. (2017) 43:56–62. doi: 10.1016/j.conb.2016.12.011 28088703 PMC5447470

[121] Berry JA Cervantes-Sandoval I Nicholas EP Davis RL. Dopamine is required for learning and forgetting in *Drosophila* . Neuron. (2012) 74:530–42. doi: 10.1016/j.neuron.2012.04.007 PMC4083655 22578504

[122] Zhou M Chen N Tian J Zeng J Zhang Y Zhang X. Suppression of GABAergic neurons through D2-like receptor secures efficient conditioning in *Drosophila* aversive olfactory learning. Proc Natl Acad Sci. (2019) 116:5118–25. doi: 10.1073/pnas.1812342116 PMC6421402 30796183

[123] Srivastava DP Esther JY Kennedy K Chatwin H Reale V Hamon M. Rapid, nongenomic responses to ecdysteroids and catecholamines mediated by a novel *Drosophila* G-protein-coupled receptor. J Neurosci. (2005) 25:6145–55. doi: 10.1523/JNEUROSCI.1005-05.2005 PMC6725065 15987944

[124] Kokay I Ebert PR Kirchhof BS Mercer AR. Distribution of dopamine receptors and dopamine receptor homologs in the brain of the honey bee, *Apis mellifera* L. Microsc Res Tech. (1999) 44:179–89. doi: 10.1002/(SICI)1097-0029(19990115/01)44:2/3<179::AID-JEMT9>3.0.CO;2-K 10084824

[125] Mustard JA Blenau W Hamilton IS Ward VK Ebert PR Mercer AR. Analysis of two D1-like dopamine receptors from the honey bee *Apis mellifera* reveals agonist-independent activity. Mol Brain Res. (2003) 113:67–77. doi: 10.1016/S0169-328X(03)00091-3 12750008

[126] Mustard JA Pham PM Smith BH. Modulation of motor behavior by dopamine and the D1-like dopamine receptor AmDOP2 in the honey bee. J Insect Physiol. (2010) 56:422–30. doi: 10.1016/j.jinsphys.2009.11.018 PMC2834802 19945462

[127] Beggs KT Hamilton IS Kurshan PT Mustard JA Mercer AR. Characterization of a D2-like dopamine receptor (AmDOP3) in honey bee, Apis mellifera. Insect Biochem Mol Biol. (2005) 35:873–82. doi: 10.1016/j.ibmb.2005.03.005 15944083

[128] Meller E Bohmaker K Goldstein M Friedhoff AJ. Inactivation of D1 and D2 dopamine receptors by N-ethoxycarbonyl-2-ethoxy-1, 2-dihydroquinoline *in vivo*: selective protection by neuroleptics. J Pharmacol Exp Ther. (1985) 233:656–62. doi: 10.1016/S0022-3565(25)22858-0 2861276

